# Supplementary material for: Stem Extract from Momordica cochinchinensis Induces Apoptosis in Chemoresistant Human Prostate Cancer Cells (PC-3)
Source: Molecules. 2022 Feb 15;27(4):1313. doi: 10.3390/molecules27041313 (PMC8878451; doi:10.3390/molecules27041313)

molecules

MDPI

Supplementary Material

Stem extract from *Momordica cochinchinensis* induces apoptosis in chemoresistant human prostate cancer cells (PC-3)

Seksom Chainumnim [1], Audchara Saenkham [2], Kulvadee Dolsophon [2], Kittipong Chainok [3], Sunit Suksamrarn [2,*] and, Wanlaya Tanechpongtamb [1,*]

[1] Department of Biochemistry, Faculty of Medicine, Srinakharinwirot University, Bangkok 10110; seksom.chainumnim@g.swu.ac.th (S.C.)
[2] Department of Chemistry and Center of Excellence for Innovation in Chemistry, Faculty of Science, Srinakharinwirot University, Bangkok 10110, Thailand; Audchara.sk@gmail.com (A.S.); kulvadee@g.swu.ac.th (K.D.)
[3] Materials and Textile Technology, Faculty of Science and Technology, Thammasat University, Pathum Thani 12121, Thailand; kchainok10@gmail.com (K.C.)
* Correspondence: wanlaya@g.swu.ac.th (W.T.); Tel.: +66813446669; sunit@g.swu.ac.th (S.S.)

Supplementary Figure S1. ^{1}H-NMR spectrum of α-spinasterol (**1**) in $CDCl_3$.

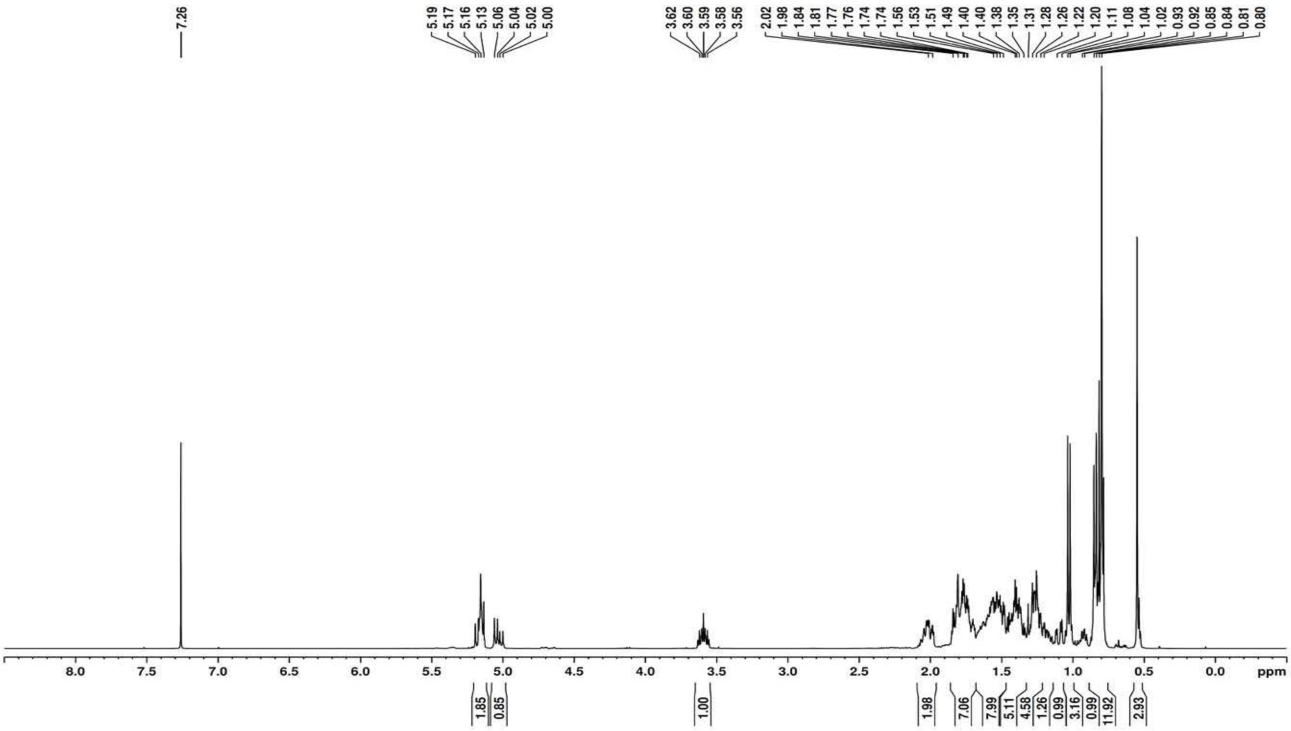

Supplementary Figure S2. ^{13}C-NMR spectrum of α-spinasterol (**1**) in $CDCl_3$.

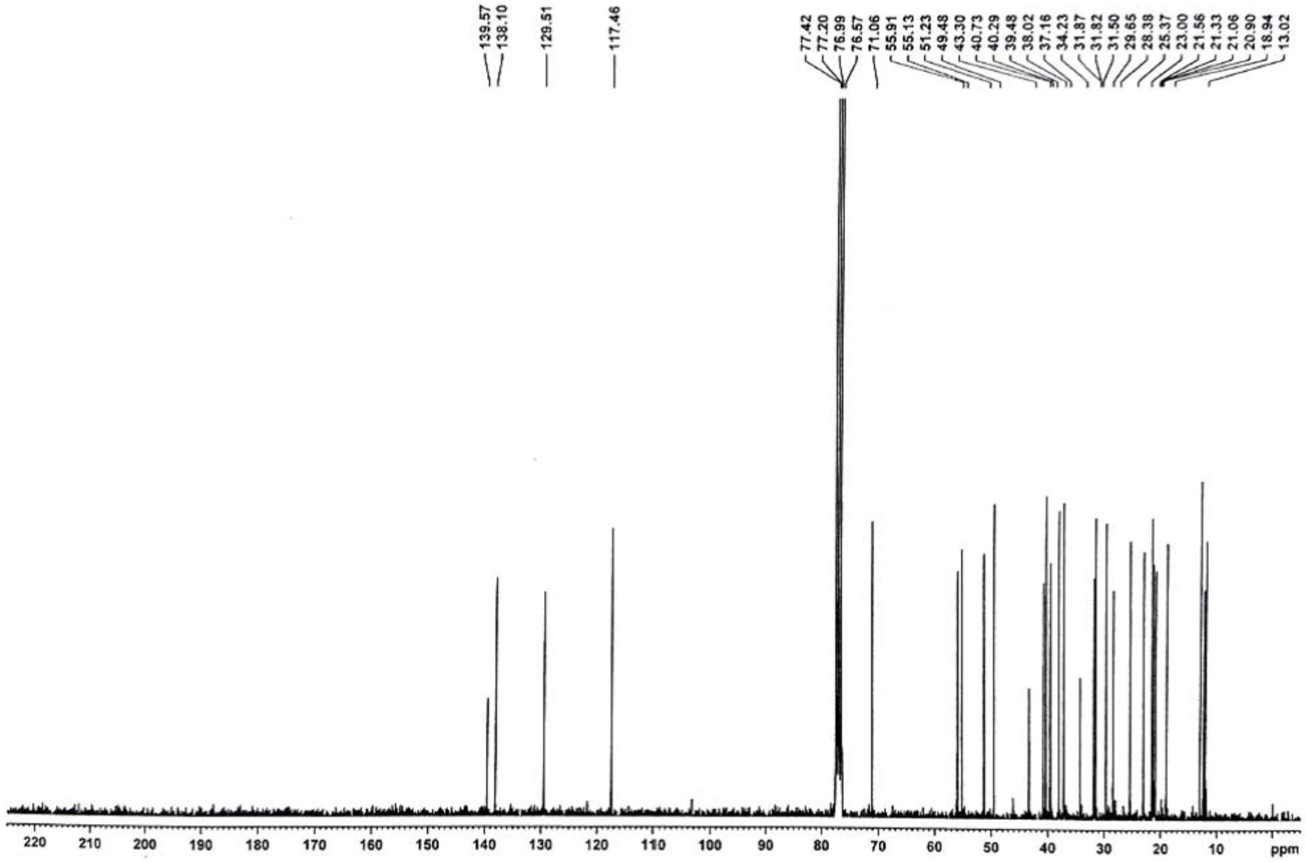

Supplementary Figure S3. ^{1}H-NMR spectrum of ligballinol (**2**) in $CDCl_3$ + DMSO-d_6.

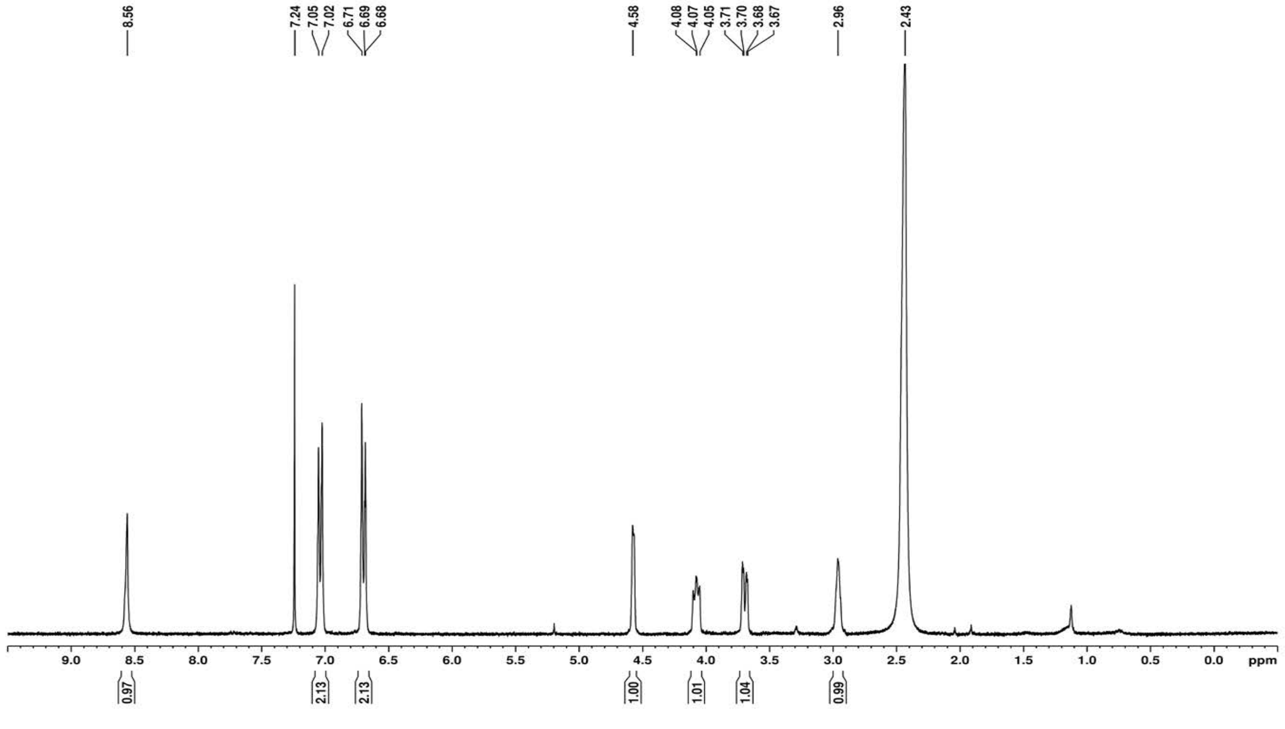

Supplementary Figure S4. ^{13}C-NMR spectrum of ligballinol (**2**) in $CDCl_3$ + DMSO-d_6.

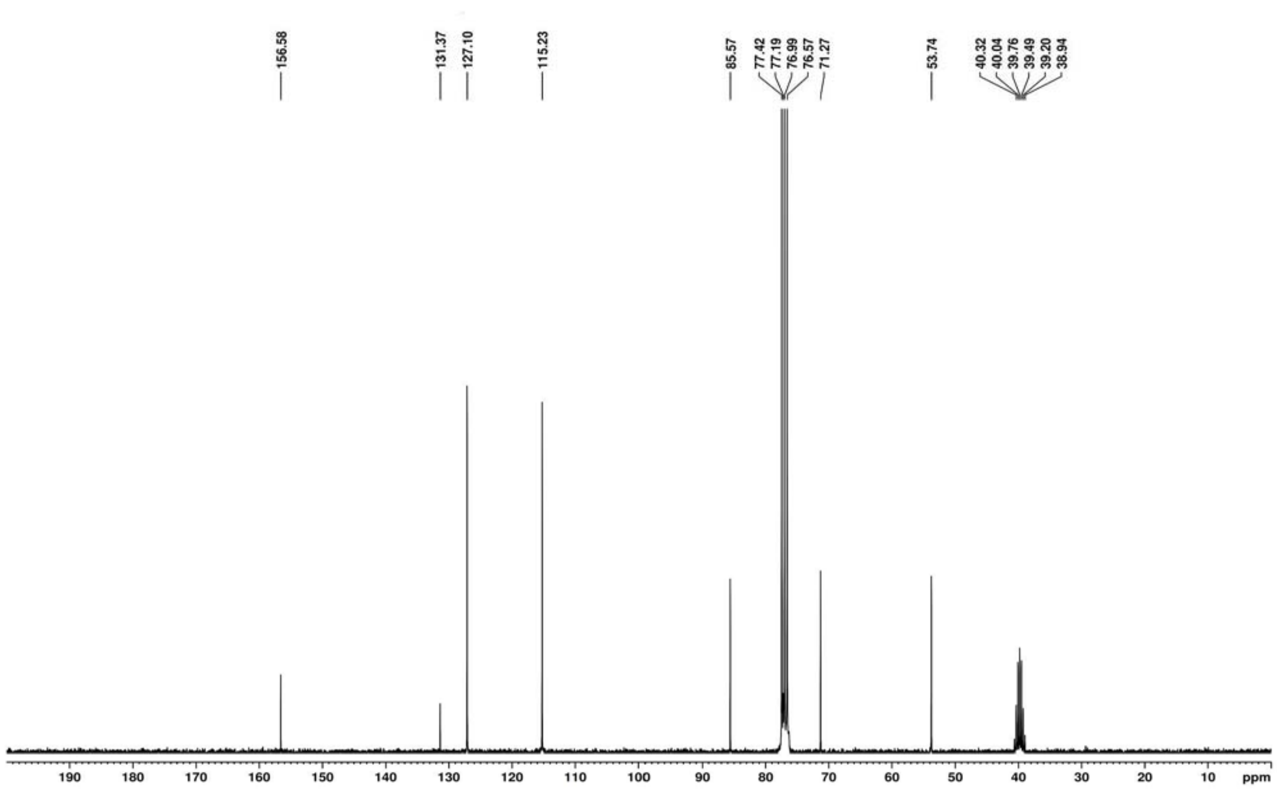

Supplementary Figure S5. HR-TOFMS spectrum of α-spinasterol.

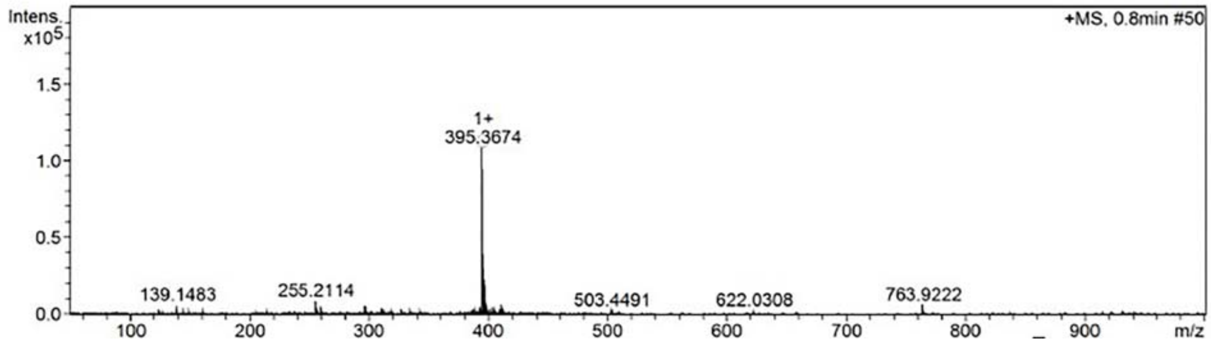

Supplementary Figure S6. HR-TOFMS spectrum of ligballinol.

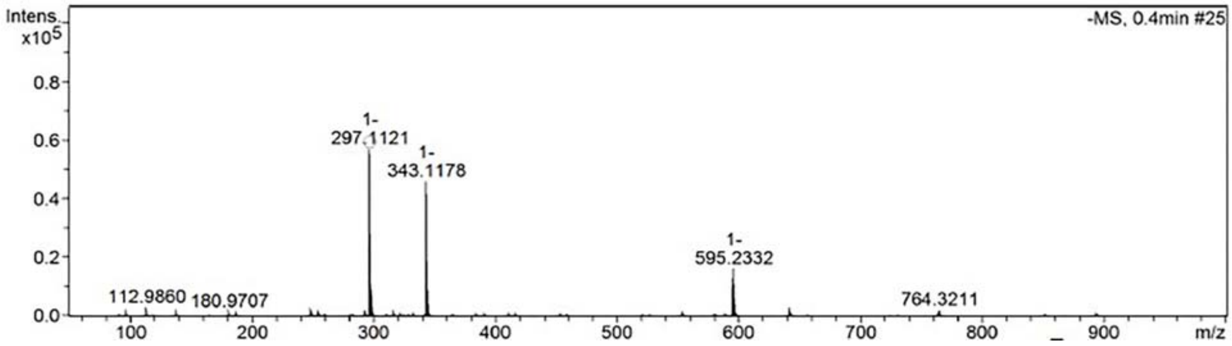

Supplement: Supplementary file 1 [file molecules-27-01313-s001.zip › molecules-1603410-supplementary.pdf]
